# Evaluation of Blood Culture Results in Patients with Malignancy in Erzurum Province, Turkey

**DOI:** 10.15388/Amed.2024.31.1.17

**Published:** 2024-02-27

**Authors:** Osman Aktas, Ozgür Akbaba, Muhammet Hamidullah Uyanik, Hakan Uslu

**Affiliations:** 1 Department of Medical Microbiology, Faculty of Medicine, Atatürk University, Erzurum, Turkey

**Keywords:** Antimicrobial susceptibility, bacteremia, blood culture, cancer, Turkey, Atsparumas antimikrobinėms medžiagoms, bakteremija, kraujo kultūra, vėžys, Turkija

## Abstract

**Background:**

Bloodstream infections are a serious public health problem that requires follow-up with blood culture; this negatively affects the course of the disease and patient healthcare costs in patients with malignancy. This study aimed to determine the growth frequency of pathogens and their antibiotic resistance profiles in the blood cultures of patients with hematological and oncogenic malignancies.

**Materials and methods:**

The results of 7451 blood cultures, obtained from 2926 patients between January 2017 and January 2022, were evaluated retrospectively. Of these cultures, 3969 were obtained from patients with malignancy (diagnostic codes C00-D48 in ICD-10) and 3482 from patients without malignancy. The hospital information management system modules were used to acquire patient data and blood culture results.

**Results:**

Various microorganisms grew in 10.1% of blood cultures. Of these organisms, 64.1% were isolated from cases of malignancy. Of the pathogens, 49.2% were gram-negative bacteria, 47.7% were gram-positive bacteria, and 3.1% were fungi. The most frequently isolated bacteria were methicillin-resistant coagulase-negative staphylococci (3.2%), *Escherichia coli* (2.3%), *Klebsiella pneumoniae* (1.0%), methicillin-sensitive coagulase-negative staphylococci (0.7%), and *Staphylococcus aureus* (0.6%). Pathogen positivity was highest in the patient cultures with urinary system cancer (23.9%), thyroid and other endocrine gland cancers (20.6%), female and male genital organ cancers (18.2%/16.9%), and digestive organ cancer (14.2%). Gram-negative bacteria to ampicillin, piperacillin, and sulfamethoxazole-trimethoprim and Gram-positive bacteria to penicillin, erythromycin, and sulfamethoxazole-trimethoprim were highly resistant. Combined resistance to imipenem and meropenem was observed in 25 Gram-negative bacteria. Twelve (48%) of the carbapenem-resistant bacteria were isolated from patients with lymphoid, hematopoietic, and related tissue malignant neoplasia.

**Conclusion:**

This study reported microorganisms and their antimicrobial resistance in the blood cultures of malignant patients, a special patient group. It pointed out that the antibiotic resistance of *Staphylococcus, Klebsiella pneumoniae*, and *E. coli* is high enough to cause problems in the treatment of patients with malignancy.

## Abstract (it)

**Įvadas:**

Kraujo infekcijos yra rimta visuomenės sveikatos problema, kurią reikia stebėti atliekant kraujo pasėlio tyrimus; tai neigiamai veikia piktybiniais navikais sergančių pacientų ligos eigą ir sveikatos priežiūros išlaidas. Šiuo tyrimu siekta nustatyti patogenų augimo dažnumą ir jų atsparumo antibiotikams profilius pacientų, sergančių hematologiniais ir onkogeniniais piktybiniais navikais, kraujo kultūrose.

**Medžiaga ir metodai:**

Retrospektyviai įvertinti 7451 kraujo pasėlio, gauto iš 2926 pacientų nuo 2017 m. sausio mėn. iki 2022 m. sausio mėn. Iš šių pasėlių 3969 buvo gauti iš pacientų, sergančių piktybiniais navikais (diagnostiniai kodai C00-D48 pagal TLK-10), ir 3482 - iš pacientų, nesergančių piktybiniais navikais. Pacientų duomenims ir kraujo pasėlio rezultatams gauti buvo naudojami ligoninės informacinės valdymo sistemos moduliai.

**Rezultatai:**

Įvairūs mikroorganizmai išaugo 10,1 % kraujo kultūrų. Iš šių organizmų 64,1 % buvo išskirti iš piktybinių susirgimų atvejų. Iš visų sukėlėjų 49,2 % buvo gramteigiamos bakterijos, 47,7 % – gramteigiamos bakterijos, o 3,1 % - grybeliai. Dažniausiai išskirtos šios bakterijos: meticilinui atsparūs koagulazei neigiami stafilokokai (3,2 %), Escherichia coli (2,3 %), Klebsiella pneumoniae (1,0 %), meticilinui jautrūs koagulazei neigiami stafilokokai (0,7 %) ir Staphylococcus aureus (0,6 %). Didžiausias patogenų pozityvumas buvo pacientų, sergančių šlapimo sistemos vėžiu (23,9 %), skydliaukės ir kitų endokrininių liaukų vėžiu (20,6 %), moterų ir vyrų lyties organų vėžiu (18,2 %/16,9 %) ir virškinimo organų vėžiu (14,2 %), kultūrose. Gramteigiamos bakterijos ampicilinui, piperacilinui ir sulfametoksazol-trimetoprimui ir gramteigiamos bakterijos penicilinui, eritromicinui ir sulfametoksazol-trimetoprimui buvo labai atsparios. Kombinuotas atsparumas imipenemui ir meropenemui nustatytas 25 gramteigiamoms bakterijoms. Dvylika (48 %) karbapenemams atsparių bakterijų buvo išskirtos iš pacientų, sergančių limfoidinių, kraujodaros ir giminingų audinių piktybiniais navikais.

**Išvada:**

Šiame tyrime aprašyti mikroorganizmai ir jų atsparumas antimikrobinėms medžiagoms piktybiniais navikais sergančių pacientų - ypatingos pacientų grupės - kraujo kultūrose. Atkreiptas dėmesys, kad *Staphylococcus, Klebsiella pneumoniae* ir *E. coli* atsparumas antibiotikams yra pakankamai didelis, kad sukeltų problemų gydant pacientus, sergančius piktybiniais navikais.

## Introduction

The term *cancer* describes a group of more than 100 diseases with multiple subtypes [1]. The main types of cancers include carcinomas that begin on the skin or in the surface tissues of internal organs, such as the lung, liver, breast, prostate, kidney, and large intestine; sarcomas that develop in connective tissue, joints, blood vessels, lymph vessels, cartilage, bone, and muscle; leukemias affecting the blood and bone marrow; and lymphomas that start in the lymphatic system responsible for fighting infections [2]. Cancer, which can develop in almost all organs and tissues in the body, can leave that body vulnerable to infection. It affects the host’s immune system for reasons such as the development of tumors, radiation therapy used in cancer treatment, cytotoxic drugs, neutropenia, or by causing malnutrition. It can increase the risk of infection in patients by disrupting anatomical barriers and facilitating the entry of bacteria into the bloodstream [3]. As a result, infections are common in patients with weakened immune systems and cancer patients who have undergone surgery, leading to prolonged hospitalization, increased healthcare costs, decreased quality of life, and decreased survival [4–6].

Bloodstream infections (BSI), which frequently occur in cancer cases, contribute to a large number of deaths [7, 8]. It is stated that the prevalence of BSI varies between 11% and 38% and increases the burden of morbidity and mortality among cancer patients [9]. It is known that approximately 200,000 cases of BSI are identified every year worldwide, and mortality rates due to BSI vary between 20% and 50% [10]. BSI can cause life-threatening organ and tissue damage by overactivating the immune system [11]. Blood cultures are taken from patients to confirm and understand whether such systemic infections are caused by bacteria or fungi [12]. A blood culture is a microbiological procedure that can determine the prevalence of infectious pathogens in an area and provide the opportunity to choose the best treatment for the patient. BSI is among the infections that cause the most serious pathologies, especially in intensive care patients [13]. Although most childhood CAs are preventable, those with bacterial BSI are at high risk of death [14].

BSI poses a risk for intensive care patients, cancer patients, patients using invasive devices such as vascular or indwelling catheters, human immunodeficiency virus (HIV) positive individuals, the elderly, and newborns [10, 14]. It is known that the risk of bacteremia is high in oncology patients [15–17]. Loss of resistance and decline in immune systems that occur in cancer cases are important factors that predispose individuals to infectious diseases. In such cases, it is critical to identify the infection and treat the patient as soon as possible. The way to effectively treat patients with BSI is to isolate the agent that causes the infection and determine the antibiotic resistance profiles of this pathogen. Our aim in this study was to determine the frequency of occurrence of microorganisms that cause BSI in oncogenic patients and their resistance profiles against antibiotics used in the treatment of these pathogens. We also aimed to contribute to the literature on the epidemiology of BSI in malignant cases.

## Materials and Methods

### Patients and Data Collection

During the five-year period from January 2017 to December 2022, the blood culture results of 7451 patients, including 3966 with various malignancies containing one of the C00–D48 diagnosis codes in ICD-10 and 3485 without any reported malignancy, were retrospectively reviewed in adult and pediatric patients. Clinical samples were sent from Atatürk University Research and Application Hospital Hematology, Medical Oncology, Radiation Oncology, and Bone Marrow Transplantation Clinics. The blood culture results and patient information were obtained electronically using hospital information management system modules. Blood cultures taken within seven days after the first positive blood culture record were defined as follow-up blood cultures. If the same person was hospitalized more than once, exceeding 30 days, each hospitalization was evaluated as a separate case. Only the first of the antibiogram results of the same bacterial species grown within 30 days for the same patient was included in the study. As a result, 7451 blood cultures were obtained from a total of 2926 patients, 1746 of whom were malignant and 1180 were nonmalignant.

### Testing and Evaluating Results

Bact/Alert FA Plus blood culture bottles for adults and Bact/Alert PF Plus blood culture bottles for children were used in blood culture processes in our laboratories, and the cultures were incubated in the BACT/ALERT 3D automatic blood culture system (bioMérieux, France). During the seven-day incubation period, positive alarm samples were transferred to 5% blood agar, chocolate agar, and eosin-methylene blue (EMB) agar medium, and the microorganisms that grew after 24-48 hours of incubation at 37°C were identified using traditional methods. The Vitek 2 automation system was utilized in our laboratory for diagnosing difficult-to-identify microorganisms and conducting antibiotic sensitivity procedures. The growth of the same organism in at least one of the repeated cultures seven days after the initial bacteremia was defined as persistent bacteremia [18]. Coagulase-negative staphylococci (CoNS), diphtheroids, *Bacillus* spp., *Propionibacterium* spp., Viridans group streptococci, *Aerococcus* spp., and *Micrococcus* spp. CDC criteria were taken into account to determine whether the strains were true bacteremia agents [19]. According to these criteria, bacteria identified in a single blood sample were considered contaminants. As part of the study, the first of the bacterial strains that grew in at least two of the repeated cultures from the same patient was used.

### Antibiogram Process

The antibiotic sensitivity of bacteria was determined by the disk diffusion method on Mueller–Hinton agar plates, and sensitivity zone diameters were interpreted in accordance with EUCAST (European Committee on Antimicrobial Susceptibility Testing) criteria [20]. We investigated the resistance of Gram-negative bacteria (GNB) to amikacin (AMI), ampicillin (AMP), ceftazidime (CAZ), ciprofloxacin (CIP), ceftriaxone (CRO), cefuroxime (CXM), cefepime (FEP), gentamicin (GEN), imipenem (IPM), meropenem (MEM), trimethoprim/sulfamethoxazole (SXT), and piperacillin/tazobactam (TZP).The resistance of Gram-positive bacteria (GPB) to AMI, CIP, clindamycin (CLI), erythromycin (ERY), fusidic acid (FUS), GEN, linezolid (LZD), penicillin (PEN), SXT, and tetracycline (TET) was investigated.

### Statistical Analysis

Microsoft Excel 2019 for Mac (Version 16.69.1) was used to create tables and graphs and perform descriptive analyses. In statistical calculations related to the patients’ ages, the date on which the cases were cultured for microbiological processing was taken into account. The mean, standard deviation (SD) and interquartile range (IQR) were used to define the numerical variable (age), which does not show a normal distribution. The chi-square (χ2) test was applied to investigate the relationship between categorical variables. If the expected frequency in at least one box in the cross-tables was less than 5, Yates’ corrected chi-square test was used instead of Fisher’s exact test.

## Results

A total of 7451 blood cultures taken from 2926 cases (1746 with malignancy and 1180 without malignancy). Table 1 gives the general characteristics of the patients according to their malignancy status. Accordingly, the blood culture results of 2410 men (mean age 43.1 years; IQR 63.3–18.2 years) and 1559 women (mean age 42.3 years; IQR 63.2–19.0 years) positive for malignancy and 1931 men (mean age 44.7 years; IQR 59.7–29.9 years) and 1551 women (mean age 40.5 years; IQR 56.9–24.2 years) negative for malignancy were evaluated. More than 1/3 (34.9%) of the patients for whom blood culture was requested were in the 45–64 age group. The number of cases in this group was significantly higher compared to the numbers of other cases (SD = 4, N = 7451, Chi square = 382.66, *p*<.00001). A blood culture was found positive in 770 of 2929 cases. However, when all cultures were taken into account, the culture positivity was 10.1%.

**Table 1:** General characteristics of the cases according to malignancy status

Features	Malignancy positive		Malignancy negative	
	Male N (%)	Female N (%)	Male N (%)	Female N (%)
**Culture–patient relationship**				
Culture (N= 7451)	2410 (32.3)	1559 (20.9)	1931 (25.9)	1551 (20.8)
Patient (N= 2926)	1050 (35.9)	696 (23.8)	669 (22.9)	511 (17.5)
**Age (years)**				
Mean	43.1	42.3	44.7	40.5
95% CI	42.11–44.1	41.1–43.53	43.81–45.59	39.48–41.51
IQR	63.3–18.2	63.2–19.0	59.7–29.9	56.9–24.2
**Age group (years)**				
0–5 (N= 566)	252 (44.5)	156 (27.6)	92 (16.3)	66 (11.7)
6–17 (N= 809)	321 (39.7)	215 (26.6)	120 (14.8)	153 (18.9)
18–44 (N= 2060)	457 (22.2)	349 (16.9)	612 (29.7)	642 (31.2)
45–64 (N= 2694)	869 (32.3)	488 (18.1)	832 (30.9)	505 (18.7)
≥65 (N= 1322)	508 (38.4)	351 (26.6)	278 (21.0)	185 (14.0)
**Blood culture results**				
Positive (N= 754)	266 (35.3)	217 (28.8)	142 (18.8)	129 (17.1)
Negative (N= 6653)	2126 (32.0)	1330 (20.0)	1779 (26.7)	1418 (21.3)
Contamination (N= 44)	18 (40.9)	10 (22.7)	12 (27.3)	4 (9.1)

The distribution of pathogen positivity according to the type of malignancy is given in Table 2. Culture positivity was significantly higher in patients with malignancies (483/3456) than in those without malignancies (271/3197) (SD = 1, N = 7407, Chi square = 39.90, *p* <.00001). The highest rate of pathogen growth was seen in cases with malignant neoplasia of the urinary tract, which includes the bladder and renal pelvis. Cases with malignancies of the thyroid and other endocrine glands ranked second, followed by malignancies of the male genital organs, female genital organs, and digestive organs, respectively. There was no growth in the blood cultures of cases with lip, oral cavity, and pharynx malignancies. The pathogen isolation rate from cases without malignancy was higher than from those with bone and joint cartilage, respiratory and intrathoracic organ, mesothelial, and soft tissue malignancies.

**Table 2:** Distribution of positive growth culture according to the site of malignancy

ICD codes (malignant neoplasm regions)	Total n	Malignancy			
		Positive		Negative	
		n	%	n	%
C00-C14 (lip, oral cavity and pharynx)	24	0	0.0	24	100.0
C15-C26 (digestive organs)	558	79	14.2	479	85.8
C30-C39 (respiratory and intrathoracic organs)	314	18	5.7	296	94.3
C40-C41 (bone and articular cartilage)	65	5	7.7	60	92.3
C43-C44 (skin melanoma and other)	20	2	10.0	18	90.0
C45-C49 (mesothelial and soft tissue)	58	1	1.7	57	98.3
C50 (breast)	116	13	11.2	103	88.8
C51-C58 (female genital organs)	77	14	18.2	63	81.8
C60-C63 (male genital organs)	71	12	16.9	59	83.1
C64-C68 (urinary tract)	46	11	23.9	35	76.1
C69-C72 (eye, brain and other parts of CNS)	99	12	12.1	87	87.9
C73-C75 (thyroid and other endocrine glands)	68	14	20.6	54	79.4
C80 (without specification of site)	436	58	13.3	378	86.7
C81-C96 (lymphoid, hematopoietic and related tissue)	1987	244	12.3	1743	87.7
Total malignancy	3939	483	12.3	3456	87.7
No malignancy	3468	271	7.8	3197	92.2
Total	7407	754	10.2	6653	89.8

CNS = Central nervous system

The results of 7386 cultures, excluding 65 cultures considered to be contamination, were examined. Taking this number into consideration, the microorganisms isolated from malignancy positive and negative cases and their isolation rates are summarized in Table 3. Of the 754 microorganisms isolated, 371 (49.2%) were GPB, 360 (47.7%) were GNB, and 23 (3.1%) were fungi. Methicillin-resistant coagulase-negative staphylococci (MR-CoNS) were most frequently isolated in GPB, followed by methicillin-sensitive coagulase-negative staphylococci (MS-CoNS) and *Staphylococcus aureus*. Among GNB, the most frequently isolated ones were *E. coli, Klebsiella pneumoniae*, and *Pseudomonas aeruginosa*. Statistical calculations in the table were made taking into account 3920 cases with malignancy and 3384 cases without malignancy. Among GPB, except for *Enterococcus* spp. and *Streptococcus* spp. strains, the prevalence of other bacteria was significantly higher in cases with malignancy than in those without malignancy. In GNB, no statistical difference could be detected between the growth rates of bacteria other than *E. coli* in malignant and nonmalignant cases.

**Table 3:** Microorganisms isolated from patients according to the presence of malignancy*

Microorganisms	Malignancy positive		Malignancy negative		Total		χ^2^	p
	n	%	n	%	n	%		
**GPB**								
MR-CoNS	155	2.1	82	1.1	237	3.2	17.917	.0000
MS-CoNS	40	0.5	12	0.2	52	0.7	13.135	.0003
*S. aureus*	33	0.4	15	0.2	48	0.6	5.492	.0191
*Enterococcus* spp.^†^	19	0.3	10	0.1	29	0.4	2.160	.1416
*Streptococcus* spp.^‡^	2	0.0	2	0.0	4	0.1	0.006	.9390
*Listeria monocytogenes*	1	0.0	-	-	1	0.0	-	-
Total GPB	250	3.4	121	1.6	371	5.0	37.694	<.0000
**GNB**								
*Escherichia coli*	113	1.5	59	0.8	172	2.3	13.445	.0002
*Klebsiella* spp. *^#^*	43	0.6	32	0.4	75	1.0	0.894	.3445
*Pseudomonas* spp.^£^	21	0.3	16	0.2	37	0.5	0.350	.5541
*Acinetobacter* spp. *^¢^*	18	0.2	16	0.2	34	0.5	0.014	.9040
*Enterobacter* spp. *^¢^*	9	0.1	9	0.1	18	0.2	0.026	.871
Other GNB ^§^	12	0.2	12	0.2	24	0.3	0.035	.8512
Total GNB	216	2.9	144	1.9	360	4.9	8.591	.0034
**Fungi** ^¢^	17	0.2	6	0.1	23	0.3	4.476	.0344
Total positive culture	483	6.5	271	3.7	754	10.2	40.137	<.0000
Negative culture	3456	46.7	3197	43.2	6653	89.8		

* Percentages were calculated based on the total number of uncontaminated cultures (7407).

† *E. faecium* (9 strains), *E. faecalis* (1 strain)

‡ *S. pneumoniae* (3 strains), *S. agatactiae* (1 strain)

*# K. pneumoniae* (70 strains), *K. oxytoca* (2 strains), others (3 strains)

£ *P. aeruginosa* (21 strains), *P. stutzeri* (2 strains), *P. fluorescens* (1 strain), *P. putida* (1 strain), others (12 strains)

*¢ A. baumannii* (18 strains), *A. lwoffii* (3 strains), *A. haemolyticus* (1 strain), others (12 strains)

*¶ E. aerogenes* (12 strains), *Enterobacter cloacae* (6 strains)

§ *Achromobacter* spp. (3 strains), *Stenotrophomonas maltophilia* (4 strains), *Burkholderia* spp. (3 strains), *Sphingomonas paucimobilis* (5 strains), *Citrobacter* spp. (4 strains), *Proteus* spp. (1 strain), *Brevundimonas dimunuta* (1 strain), *Rhizobium radiobacter* (1 strain)

¢ *Candida* spp. (15 strains), *Candida albicans* (8 strains)

**Figure 1:**
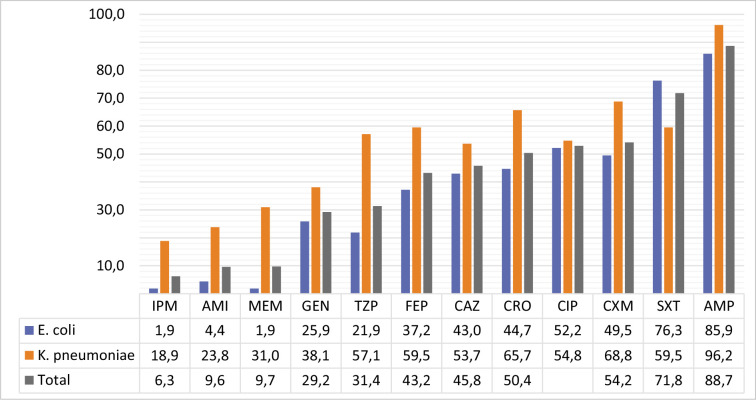
Percentages of resistance to antibiotics in GNB

Figure 1 gives the antibiotic resistance rates of GNB (114 *E. coli* and 42 *K. pneumoniae*) isolated from patients with malignancy. Since other GNB were isolated in low numbers, susceptibility results for these bacteria were not given. The highest antibiotic resistance is against AMP, SXT, and CIP in *E. coli* strains, respectively; it was observed against AMP, CXM, and CRO in *K. pneumoniae* strains. It was determined that the most effective antibiotics against these two bacteria were IPM, AMI, MEM, and GEN. A total of 25 GNB, including nine *Acinetobacter* spp., eight *Klebsiella* spp., seven *Pseudomonas* spp., and one *E. coli* strain, showed resistance to IMP and MEM together. Twelve (48%) of the carbapenem-resistant bacteria were found in people who had “lymphoid, hematopoietic, and related tissue malignant neoplasia” (ICD code C81–C96), and six (24%) were found in people who had “digestive organ malignant neoplasia” (ICD code C81–C96). We did not include the antibiogram results of nonfermentative bacteria in this study because we thought that they could not fully reflect the true resistance rates because they grow in low numbers.

**Figure 2:**
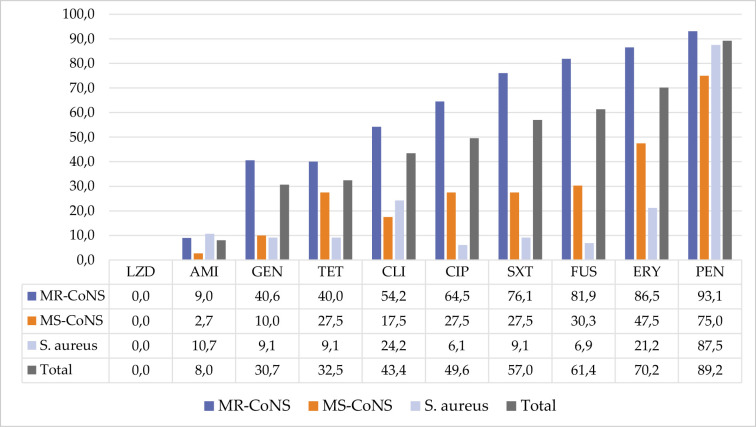
Percentages of resistance to antibiotics in GPB

The antibiotic resistance profiles of GPB are shown in Figure 2. Among GPB, the highest resistance was seen against PEN and ERY in the MR-CoNS, and the most effective antibiotics were LZD, AMI, GEN, and TET. FUS was among the most effective antibiotics, after LZD and CIP, for *S. aureus* strains. However, it remained among the antibiotics with the lowest effect on CoNS strains.

## Discussion

Atatürk University Faculty of Medicine Research Hospital, where the study was conducted, serves a population of approximately 4.5 million in 13 provinces of northeastern Anatolia, including Erzurum and its surroundings: Kars, Iğdır, Ağrı, Ardahan, Erzincan, Bayburt, Artvin, Gümüşhane, Muş, Bingöl, Van, and Malatya. With this capacity, we can say that the frequency of bacteria growing in blood cultures and the antibiotic resistance profiles of cancer patients in our hospital are representative of the region.

The frequency of patients with serious diseases such as cancer receiving inpatient treatment due to both community-acquired infections and “healthcare-associated infections (HAIs)” is high. Central venous catheters, which are frequently used for treatment and parenteral nutrition in cancer cases, create a high risk of infection [21–23]. During the five-year period, 4460 of 7386 patients (60.4%) were admitted to our hospital at least twice and were hospitalized. In these cases, different bacteria were observed to grow in blood cultures taken at different admission times. These results show that oncogenic patients with infections are likely to be re-infected with the same species of microorganism or a different type of microorganism at later times, even if they recover during treatment.

In our region, the number of blood culture-positive oncogenic male patients was higher in all age groups except those aged 75 and over. A significant relationship was observed between age and gender variables in our cases. This result is compatible with Turkish cancer data reported for gender in 2018. In the report of the Ministry of Health of the Republic of Turkey, aging is one of the main factors in the development of cancer. It has been reported that the age-standardized cancer rate is higher in men than in women (262.4/188) [24]. A recent study reported that the annual incidence of BSI in age groups over 65 years of age is more than 6,000 per 100,000 people [25]. In this study, it was stated that more than half of BSIs occurred in people aged 65 and over, and 70% of the deaths were in this age group, indicating that BSIs are an important problem for the elderly. Uslan et al. reported that there are significant differences in the distribution of organisms in patients with BSI according to age and gender, that the incidence of BSI increases rapidly with age, and that it is more common in men than in women [26]. It is possible to say that the low number of elderly cases is due to Erzurum’s young population. According to 2015 data, only 3.2% of the total population in Erzurum is 75 years old or older [27].

Apart from bacteria, only *Candida* spp., a fungal species, grew in the blood cultures of the patients. The diversity of aerobic and facultative anaerobic bacteria from different genera that cause BSIs was found to be high in our region. GNB were isolated from blood cultures more frequently than GPB. *E. coli* is the most frequently isolated GNB, followed by *K. pneumoniae* and *P. aeruginosa* strains. CoNS are the most isolated microorganisms among GPB. It is known that CoNS are the most frequently contaminating organisms in blood cultures. However, the presence of this bacterium in at least two or more repeat cultures taken during the same time period indicates that the majority of them will not be contaminated. Jayaweera and Sivakumar [23] reported that 19.2% of BSI agents associated with central venous catheters in children were CoNS strains. They also reported that CoNS bacteremia was significantly associated with these asymptomatic cases. The second most prevalent GPB in our region was MS-CoNS, followed by *S. aureus* and *Enterococcus* spp. Baier and colleagues [14] showed that GPB are more common than GPB in patients with central venous catheters with various types of cancer, such as acute myeloid leukemia, non-Hodgkin lymphoma, and acute lymphoblastic leukemia. According to Amanati et al. [7], adult patients in Iran suffering from malignant illness had higher rates of both GPB and GNB in their BSI. The most common pathogens detected in this Iranian study were *E. coli* and CoNS. In Spain, Royo-Cebrecos and colleagues [28] found that the most common etiological agents in polymicrobial BSIs were GNB, and *E. coli* strains were particularly common. Siddiqui et al. [29] identified *S. aureus, E. coli, Pseudomonas* spp., and *Klebsiella* spp. as the most common BSI agents in febrile neutropenic patients in Pakistan. The majority of other sources in the literature all agree that staphylococci from GPB and *E. coli* from GNB were more often recovered from blood samples. This feature was also observed in our study.

Resistance of bacteria to antimicrobials is a global health problem that complicates the management of infectious diseases. It is stated that significant increases in the resistance of Enterobacteriaceae members to third-generation cephalosporins, aminoglycosides, fluoroquinolones, and aminoglycosides have been observed [30]. Members of *Enterobacteriaceae* that produce oxacillinase, carbapenemase, and metallo-β-lactamase enzymes threaten the effectiveness of carbapenems [30, 31]. Members of *Enterobacteriaceae* and nonfermentative GNB are inherently resistant to benzylpenicillin, fusidic acid, macrolide, lincosamide, streptogramin, rifampicin, daptomycin, and linezolid [32]. Nonfermentative GNB infections such as *P. aeruginosa, A. baumannii*, and *Stenotrophomonas maltophilia*, which require medical and surgical procedures, can be life-threatening, especially in immunocompromised patients [33]. The most effective antibiotics against *E. coli* and *K. pneumoniae* isolated from our region were carbapenems (IPM and MEM) and AMI, one of the aminoglycosides. However, the resistance rate of *Klebsiella pneumoniae* strains to these antibiotics ranges from 18.9% to 55.6%. The total resistance rates of these two bacteria to other antibiotics vary between 35.7% and 86.7%. Apart from the fact that the resistance of *E. coli* strains isolated from malignancy-negative cases to carbapenems was higher than that of those isolated from malignancy-positive cases, such a relationship could not be established for other antibiotics.

It has been stated that new drugs may need to be developed for the treatment of multidrug-resistant bacteria such as methicillin-resistant *S. aureus*, and vancomycin-resistant *E. faecium* among GPB [34]. The source of these concerns is antibacterial resistance, acquired through genetic mutations or acquired genomes. CoNSs are increasingly associated with healthcare-associated infections (HAI) in those with compromised immune systems and those using invasive medical devices [35, 36]. CoNSs, which are responsible for 30–40% of nosocomial BSIs, are bacteria that can acquire resistance to routinely used antibiotic classes including beta-lactams, aminoglycosides, and macrolides, as well as last-resort antibiotics like glycopeptides [37]. Among CoNSs, *S. epidermidis* is a bacterium that frequently infects prosthetic devices and IV catheters, posing a risk of BSI [38]. More than half of the GPB consisting of MR-CoNS, MS-CoNS, and *S. aureus* strains isolated from patients in the Erzurum region were resistant to PEN, ERY, FUS, and SXT. It has been observed that resistance rates of this bacterial community to antibiotics (GEN, TET, CLI, and CIP) other than LZD and AMI, which are the most effective antibiotics, are over 28%.

In cases of severe sepsis, appropriate empiric treatment may be required without obtaining blood culture results due to the possibility of losing patients if treatment is delayed [39]. Early, rapid, and effective antimicrobial treatment plays a key role in the recovery of patients, especially those with the criteria for sepsis or septic shock [40]. However, it is known that increasing antibiotic resistance and the natural delay between taking blood samples for culture and determining the in vitro sensitivity of isolated bacteria pose problems in empirical antibiotic selection [41]. Factors such as prolonged hospital stays, surgical invasive treatment practices, mechanical ventilation, weakening of the immune system, recent surgery, and the use of catheters play a role as major risk factors in the development of bacterial infections [38, 42].

Bacterial infections that develop in neutropenic patients with weak immune response and a high risk of developing sepsis should be treated as soon as possible with an appropriate antibiotic. It is stated that empirical antibacterial therapy should include a bactericidal, well-tolerated β-lactam (piperacillin-tazobactam, cefepime, imipenem-cilastatin, or meropenem) with activity against GPB and GNB organisms, including *P. aeruginosa*. Again, while it is recommended that empirical treatment with cefepime or piperacillin-tazobactam against ESBL-producing GNB be expanded to include imipenem-cilastatin or meropenem, it is also recommended that vancomycin, daptomycin, and linezolid, which have activity against resistant Gram-positive cocci such as MRSA and vancomycin-resistant enterococci (VRE), should not be included in the initial empirical treatment regimen [43].

Because our study was based on patient samples, nosocomial BSI rates could not be determined. Additionally, we did not have any information about the antibiotics used in the treatment of the patients or the success of the treatment. These situations were the most important limitations of the study.

## Conclusions

As a result, a wide spectrum of pathogens, including different genera and species, were isolated from the blood cultures of patients with malignancy in our region. These bacteria were also found to be highly resistant to the antibiotics used in treatment, so much so that antibiotics are unlikely to be effective, except for IMP, AMI, and MEM in GNB and LZD and AMI in GPB. Which antibiotics will be given in the empirical treatment of infections that develop in patients with a serious disease such as cancer should be decided in cooperation with the patient’s physician and the infection control committee. Most importantly, the rapid diagnosis and treatment of BSIs are very important in reducing patient deaths, hospital stays, and patient care costs.

First of all, rapid diagnosis and rapid treatment of BSIs are very important in reducing patient deaths, hospital stays, and patient care costs.
